# Editorial: The Earliest-Born Cortical Neurons as Multi-Tasking Pioneers: Expanding Roles for Subplate Neurons in Cerebral Cortex Organization and Function

**DOI:** 10.3389/fnana.2020.00043

**Published:** 2020-08-25

**Authors:** Makoto Sato, Shen-Ju Chou

**Affiliations:** ^1^Department of Anatomy and Neuroscience, Graduate School of Medicine, Osaka University, Suita, Japan; ^2^Division of Developmental Neuroscience, United Graduate School of Child Development, Osaka University, Suita, Japan; ^3^Institute of Cellular and Organismic Biology, Academia Sinica, Taipei, Taiwan

**Keywords:** cerebral cortex, development, layer 6b, neocortex, subplate

The subplate is a unique layer in the mammalian neocortex. It consists of the earliest-born neurons in the neocortex and undergoes a massive reduction in the number of neurons during postnatal development. The subplate is also characterized by its heterogeneity in cell morphology, incomparable gene expression pattern, and an early functional maturation. This diversity in form and function is evident in its role in circuit formation processes between the cortex and thalamus and also within a local cortical area above it. Disruptions of the subplate can lead to neurodevelopmental deficits such as autism spectrum disorder.

Postmitotic neurons born in the ventricular zone first form the preplate together with Cajal-Retzius cells, which originate from three distinct regions of the dorsal telencephalon (reviewed in Barber and Pierani, 2016). The preplate is then split, by later-born neurons coming in between to form the cortical plate, into the marginal zone at the surface (containing Cajal-Retzius cells) and the subplate at the base of the cortical plate. Although most of the subplate neurons are early born preplate neurons in rodents, the vast majority of subplate neurons are generated during mid-gestation in primates (Duque et al., 2016). While many subplate neurons are lost during postnatal development, not all of them disappear. Recent morphological and gene expression studies have provided evidence that layer 6b in the adult (or juvenile) cortex contains remnant subplate neurons (Hoerder-Suabedissen et al., 2013; Marx et al., 2017). Subplate neurons exhibit morphological heterogeneity in the somatodendritic structure. In addition to typical pyramidal neurons found in layers 2-6a; horizontal cells, multipolar cells, inverted pyramidal cells, fusiform cells, and polymorphous cells are among those reported in the subplate (Mrzljak et al., 1988; Hanganu et al., 2002). These cell types are maintained between the early postnatal subplate and juvenile layer 6b despite a decrease in their abundance (Marx et al., 2017). The subplate is more than a transient embryonic structure. In primates, the subplate is much thicker, and subplate neurons remain as “interstitial cells” in the white matter (Kostovic and Rakic, 1990), suggesting prominent roles of subplate neurons in primates. As pyramidal neurons increase in abundance in layer 6b, it is suggested that layer 6b consists of remnant subplate neurons and cortical pyramidal neurons. Interestingly, intermediate progenitor cells expressing *Tbr2* contribute to projection neurons in all layers, including preplate Cajal-Retzius neurons and the subplate (Vasistha et al., 2015; Mihalas et al., 2016), suggesting that pyramidal neurons that increase in juveniles might derive from *Tbr2*-expressing progenitors.

Transcriptomic analyses of cortical neurons and those focused on subplate neurons, identified genes specifically expressed in subplate/layer 6b with a differential time course (Bernard et al., 2012; Oeschger et al., 2012; Hoerder-Suabedissen et al., 2013; Tasic et al., 2016). A cell clustering analysis based on single cell RNAseq data classified layer 6b cells into two cell types (Tasic et al., 2016). Molecular markers for subplate/layer 6b neurons, however, have been shown to be expressed in overlapping populations (Hoerder-Suabedissen and Molnár, 2013; Tiong et al.), making it difficult to correlate gene expression profiles with cellular morphology, neuronal connection patterns, and electrophysiological properties.

During the embryonic stage, the subplate serves as a critical interface between cortical neurons and incoming thalamocortical axons. Thalamocortical axons transiently connect with subplate neurons before they enter the cortical plate and finally reach layer 4 (Kostovic and Goldman-Rakic, 1983; Kageyama and Robertson, 1993; Herrmann et al., 1994). This transient connection is functional, as thalamic stimulations in the thalamocortical slices from rat embryos induce responses in subplate neurons (Higashi et al., 2002; Molnár et al., 2003), indicating the early maturation of subplate neurons. Furthermore, subplate neurons contain positional cues for thalamic axons to target appropriate cortical areas. When the areal identity is disorganized by mis-expressing cortical patterning molecule FGF8, in the subplate as well as in cortical plate, thalamic axons run longer in the subplate before they turn into the cortical plate (Shimogori and Grove, 2005). On the other hand, projection to the thalamus by subplate neurons was thought to pioneer the cortico-thalamic projections by neurons in layers 5 and 6. At least in ferrets, however, axons of layer 5 neurons arrive at the thalamic nuclei earlier than those of subplate and layer 6 neurons (Clascá et al., 1995), arguing against the pioneering function of subplate axons. Additionally, the subplate (layer 6b) also shapes corticofugal pathways (Grant et al., 2012) and callosal connections (deAzevedo et al., 1997). For example, retinal inputs regulate layer 6b neuronal projections, which may in turn influence the projection of layer 5 neurons (Grant et al., 2016).

Subplate neurons are also indispensable for local network formation, especially in the primary sensory areas. For example, ablation of the subplate in the visual cortex affects ocular dominance column formation by affecting the maturation of thalamocortical connections to layer 4 (Ghosh and Shatz, 1992; Kanold and Shatz, 2006).

In addition to the foundational studies described above, recent work has revealed new aspects of the subplate function and form, such as modulation of radial migration of later-born neurons (Ohtaka-Maruyama et al., 2018), extra-cortical origins (Pedraza et al., 2014), and fate selection of later-born neurons (Ozair et al., 2018). This Research Topic entitled *The Earliest-Born Cortical Neurons as Multi-Tasking Pioneers: Expanding Roles for Subplate Neurons in Cerebral Cortex Organization and Function*, consists of a collection of three Review articles that provide up-to-date overviews on multiple functions of the subplate in cortical development and two Original Research articles that report novel findings in the development and function of the subplate.

Luhmann et al. summarize the electrophysiology of subplate neurons including intrinsic membrane properties and firing patterns, and input/output connection patterns of subplate neurons, discussing possible roles in cortical spindle burst and gamma oscillation.

A review by Kanold et al. explains sensory-evoked plasticity of neuronal circuits of subplate neurons during development and in pathological conditions, focusing on the silent synapses formed onto them.

In addition to the two Review articles above, a Mini Review by Ohtaka-Maruyama features a novel function of the subplate in the regulation of the migration of cortical plate neurons. This finding revealed another mechanism for mode switching of neuronal migration from slow multipolar migration to rapid locomotion, guided by radial fibers.

Yu et al. use conditional mouse knockouts to define new functions of a well-established subplate marker gene, *Ctgf*, in regulating the number and dendritic complexity of subplate neurons, and maturation of oligodendrocytes in the white matter beneath the primary somatosensory cortex.

Finally, an article by Tiong et al. identified a novel marker gene for the mouse embryonic subplate and shows that it is expressed in 80% of layer 6b neurons in the primary somatosensory cortex that project axons to the primary motor cortex. This marker should be a useful tool to study functions of subplate neurons at early stages of cortical development.

The aim of this Research Topic is to highlight the versatility of the subplate in cortical development and to attract readers to this unique layer in the mammalian neocortex. We would like to thank all the contributors and readers and hope future work will elucidate developmental mechanisms and circuit functions of the subplate, which is important both scientifically and clinically.

## Author Contributions

All authors listed have made a substantial, direct and intellectual contribution to the work, and approved it for publication.

## Conflict of Interest

The authors declare that the research was conducted in the absence of any commercial or financial relationships that could be construed as a potential conflict of interest.
